# Success rates of smoking cessation therapies to patients with mental illness by video consultants or by treatment in the community: A Randomized Controlled Trial

**DOI:** 10.1192/j.eurpsy.2022.471

**Published:** 2022-09-01

**Authors:** M.K. Sorensen, M. Rasmussen, P. Hjorth, R. Christiansen

**Affiliations:** 1 Psykiatrien i region syddanmark, Lokalpsykiatrien Vejle, Vejle, Denmark; 2 Region of Southern Denmark, Department Of Psychiatry Vejle, Vejle, Denmark

**Keywords:** schizophrénia, affektiv disorder, Randomized Controlled Trial, smoking cessation

## Abstract

**Introduction:**

Smoking is probably the one single factor with the highest impact on reducing the life expectancies of patients with mental illness. In Denmark, 38.8% of patients with persistent mental health problem are smoking. Patients may have problem in participating in ordinary smoking cession programs offered in the community, but they are concerned about the impact of tobacco use on their health and finances and are motivated to stop smoking.Videoconferencing addressing smoking cessation might be an alternative to ordinary consultation at the clinic because the patients can access the treatment at home.

**Objectives:**

Compare rates of smoking cessation in two interventions.

**Methods:**

Patients diagnosed with schizophrenia, bipolar disorders or depression in 3 outpatient clinics are eligible for inclusion.Primary outcome is changes in number of cigarettes smoked pr. patients per day in at 6-month follow-up. Secondary outcome is abstinence from smoking at 6-month follow-up.This is a two-arm randomized controlled trial. 1. Daily video consultants at the start of smoking cessation and the months after. 2. Treatment as usual consistent of smoking cessation treatment in the community by weekly consultants.

**Results:**

By September 2021, we have included 64 patients. Among patients, receiving video 26% has stopped and 15% has stopped from treatment as usual.Many patients has reduced their smoking considerably. The patients in general express that they are satisfied with both interventions.

**Conclusions:**

Smoking cessation delivered by daily short video consultants seems to be the best and most effective way to help patients with serious mental illness to stop smoking.

**Disclosure:**

No significant relationships.

